# Construction of a Prognosis-Related Gene Signature by Weighted Gene Coexpression Network Analysis in Ewing Sarcoma

**DOI:** 10.1155/2022/8798624

**Published:** 2022-01-27

**Authors:** Runhan Zhao, Chuang Xiong, Chao Zhang, Lin Wang, Hao Liang, Xiaoji Luo

**Affiliations:** Department of Orthopedics, The First Affiliated Hospital of Chongqing Medical University, Yuzhong, Chongqing, China

## Abstract

**Background:**

Ewing sarcoma (ES) is the second most common pediatric bone tumor with a high rate of metastasis, high recurrence, and low survival rate. Therefore, the identification of new biomarkers which can improve the prognosis of ES patients is urgently needed.

**Methods:**

Here, GSE17679 dataset was downloaded from GEO databases. WGCNA method was used to identify one module associating with OVS (overall vital survival) and event. cytoHubba was used to screen out 50 hub genes from the module genes. Then, GSE17679 dataset was randomly divided into train cohort and test cohort. Next, univariate Cox analysis, LASSO regression analysis, and multivariate Cox analysis were conducted on 50 hub genes combined with train cohort data to select pivotal genes. Finally, an optimal 7-gene-based risk assessment model was established, which was verified by test cohort, entire GSE17679, and two independent datasets (GSE63157 and TCGA-SARC).

**Results:**

The results of the functional enrichment analysis revealed that the OVS and event-associated module were mainly enriched in the protein transcription, cell proliferation, and cell-cycle control. And the train cohort was divided into high-risk and low-risk subgroups based on the median risk score; the results showed that the survival of the low-risk subgroup was significantly longer than high-risk. ROC analysis revealed that AUC values of 1, 3, and 5-year survival were 0.85, 0.94, and 0.88, and Kaplan-Meier analysis also revealed that *P* value < 0.0001, indicating that this model was accurate, which was also verified in the test, entire cohort, and two independent datasets (GSE63157 and TCGA-SARC). Then, we performed a comprehensive analysis (differential expression analysis, correlation analysis and survival analysis) of seven pivotal genes, and found that four genes (NCAPG, KIF4A, NUF2 and CDC20) plays a more crucial role in the prognosis of ES.

**Conclusion:**

Taken together, this study established an optimal 7-gene-based risk assessment model and identified 4 potential therapeutic targets, to improve the prognosis of ES patients.

## 1. Introduction

Ewing sarcoma (ES) is the second most common pediatric bone tumor with a high rate of metastasis, high recurrence, and low survival rate. Although a standard multimodal treatment regimen which includes surgical resection, local radiotherapy, and intensive multiagent chemotherapy has been established [1], 30-40% of patients still develop recurrence or metastasis after comprehensive therapy [2]. And a British cohort of patient study reported that the 5-year survival rate of patients with ES was only 55% [3]. Due to a lack of reliable statistical tools and target genes, diagnosing and treating ES patients are very challenging [4]. Therefore, further research into the pathogenesis of ES is required to improve the efficiency of diagnosis and treatment of ES.

Previous study has reported that ES is characterized by FET-ETS gene fusions [5, 6], and additional genetic alterations apart from FET-ETS fusions are exceedingly rare [7–10]. Meanwhile, Ewing sarcoma breakpoint region 1 (EWSR1) - E26 transformation-specific (ETS) fusion gene is the major factor (85% of cases are EWSR1-FLI1, and 10% of cases are EWSR1-ERG), and EWS-ETS may promote tumor metastasis and invasion by altering RNA transcriptional regulation and epigenetic modification [11, 12]. Therefore, many studies focus on EWS-ETS-positive Ewing sarcoma and revealed the unique role of this fusion gene in the development of ES. However, treatment targeting the EWS-ETS fusion gene still has been a challenge, and there is also increasing evidence demonstrating that EWS-ETS not be the sole factor in the occurrence and development of ES [13–16]. In addition, the prognostic value of the EWS-ETS fusion gene is unclear with patients of ES. A retrospective study found that EWSR1-FLI1 transcript subtypes are significantly associated with outcomes of patients [17]. But, the results of two prospective studies did not support this observation [18, 19]. Consequently, the identification of new therapeutic or predictive biomarkers which can improve the prognosis of ES patients is urgently needed.

In this study, we downloaded the gene expression profiles and clinical data from GSE17679 dataset and two independent datasets (GSE63157 and TCGA-SARC) for validation. We used the WGCNA method to identify one module associating with OVS and event. Then, univariate Cox regression analysis, LASSO regression analysis, and multivariate Cox regression analysis were used to screen out signature hub genes related to the prognosis of ES patients. Finally, we established an optimal 7-gene-based risk assessment model to evaluate the survival of ES patients. Our study provides a new method to assist the prediction of prognosis in clinical ES patients.

## 2. Materials and Methods

### 2.1. Data Collection and Preprocessing

The gene expression profiles and clinical data were downloaded the from the Gene Expression Omnibus (GEO) database (https://www.ncbi.nlm.nih.gov/geo/) as dataset GSE17679, which was based on the GPL570 platform ((HGU133 Plus 2) Affymetrix Human Genome U133 Plus 2.0 Array). And probes of the array data were replaced with the corresponding official gene symbols using the hgu133plus2.db (version 3.2.3) package. If multiple probes were mapped to the same gene symbol, duplicates were collapsed randomly. Then, 106 samples of human tissue were recognized by consensus clustering, and outlier samples were removed. Finally, a total of 88 samples were selected for the next analysis.

### 2.2. Construction of a Weighted Coexpression Network

We use the WGCNA (version 1.70-3) package in R to deal with ES data and construct a weighted coexpression network [20]. Initially, Pearson's correlation coefficient was used to construct a correlation matrix. Next, we find a most proper soft-thresholding power (*β*) in the range of 1 to 30 using pickSoftThreshold function. This *β* value can get with a balance between scale-independence and mean connectivity in a coexpression network, which was used to transform the correlation matrix into a weighted adjacency matrix. Then, the adjacency matrix was transformed into a topological overlap matrix (TOM). Based on TOM, we can get 1-TOM, which was used as the distance measurement to cluster genes into coexpression modules, and highly correlated modules which Pearson′s correlation ≥ 0.75 were merged in one. Finally, the *P* values and correlation coefficients between a coexpression module and a clinical feature were calculated and visualized by heatmaps.

### 2.3. Identification of Clinically Meaningful Modules

Module eigengene (ME, the first principal component of a given module) can represent the expression levels of all genes in a module. Gene significance (GS) was defined as the correlation between genes and clinical features in linear regression. Module significance (MS) means the average GS for all the genes in a module. In a word, MS was used to select modules having highly relation with interested clinical feature, and the selected module was used for further analysis.

### 2.4. Construction of the PPI Network and Identify Hub Genes

Retrieval of Interacting genes/Proteins (STRING) database (https://string-db.org) contains information about the interactions between proteins, which was used to construct the protein-protein interaction (PPI) network. First, we upload the module genes of interest to build the PPI network using STRING online analysis tool. Next, the PPI network was imported into the software of Cytoscape (version 3.8.2). Finally, top 50 hub genes with high Maximal Clique Centrality (MCC) values were selected using cytoHubba in Cytoscape for subsequent analysis.

### 2.5. GO and KEGG Analyses

Gene Ontology (GO) and Kyoto Encyclopedia of Genes and Genomes Pathway Enrichment (KEGG) were two important databases to analyze the function of genes. To determine the function of top 50 hub genes, the clusterProfiler R package was used to perform GO and KEGG analyses with threshold *P* < 0.05 [21].

### 2.6. Construction of Prognosis-Related Risk Assessment Model and Evaluation

First, GSE17679 datasets were randomly divided into train cohort and test cohort, with train cohort being used for construction of the risk model and test cohort and entire cohort being used as validation data. To construct a reliable model, three analysis methods were used by step: univariate Cox regression analysis-LASSO (least absolute shrinkage and selection operator) regression analysis-multivariate Cox regression analysis [22]. According to the risk model, samples in the two sub cohorts (train and test) and entire cohort were, respectively, assigned a risk score and divided into high-risk group and low-risk group based on the median risk score. Then, to estimate the sensitivity and specificity of this model, the correlation between signature gene expression and survival outcome of patients was performed, and the receiver operating characteristic (ROC) curves were drawn. Finally, two independent datasets (GSE63157 and TCGA-SARC) were also used for further validation of this model.

### 2.7. Analysis of Each Single Pivotal Prognostic Gene

First, we used boxplot to observe the expression levels of prognostic genes in the final outcome of patients and determine whether they were statistically significant. Then, we do a correlation analysis between the expression levels of the prognostic genes and clinical traits, to select more critical genes in prognosis of ES patients. Finally, the median value of gene expression for selected genes was calculated to group patients with above median value as high expressers and below the median value as low expressers. And, based on the expression group, we do survival analysis to further verify the importance of selected genes in prognosis.

### 2.8. Construction of the Nomogram and Validation

At the end of present research, clinical information on ES samples, such as gender, age, metastasis, event and survival time, was incorporated with the risk score for nomogram construction. And calibration plot was drawn to verify the accuracy of nomogram.

## 3. Results

### 3.1. Construction of a Weighted Coexpression Network

**Figure 1:**
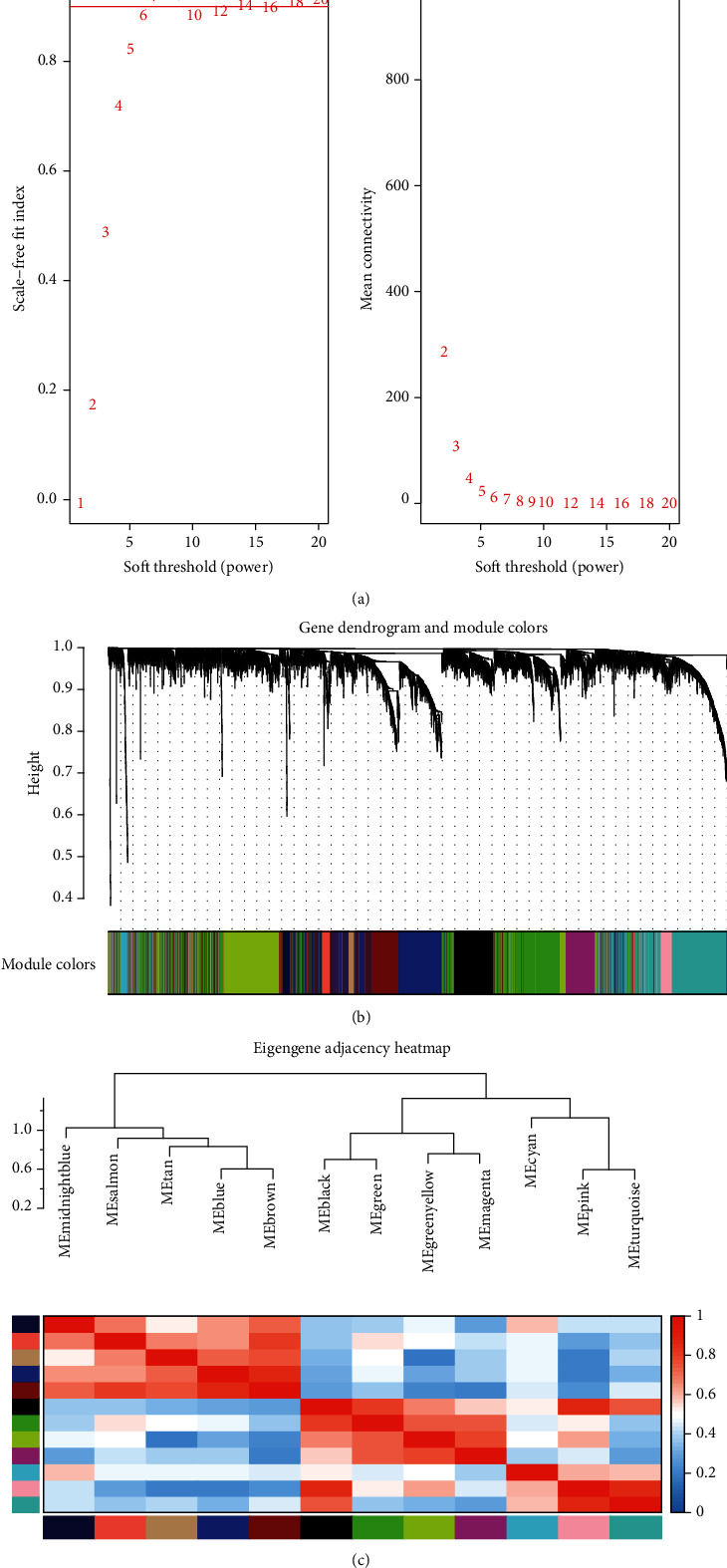
Construction of coexpression modules based on GSE17679 dataset by WGCNA. (a) Determining soft-thresholding power in WGCNA: the scale-free fit index and the mean connectivity for various soft-thresholding powers. (b) The cluster dendrogram and color display of coexpression network modules. The different colors correspond to the coexpression modules in Ewing sarcoma. (c) Hierarchical clustering of module hub genes and heatmap plot of the adjacencies in the hub gene network.

We downloaded the expression matrix and clinical information of GSE17679 from the GEO database and calculated the scale-independence and the mean connectivity at different thresholds. A suitable soft threshold 6 was chosen, which met the standard which scale − independence > 0.85 and average connectivity < 100 (Figure 1(a)). Under the soft-thresholding power of 6, cut height of 0.25, and minimal module size as 30, coexpression network was constructed and 12 modules were identified (Figure 1(b)). And there is presently acceptable discriminability between each module in the similarity heatmap plot (Figure 1(c)).

### 3.2. Identification of Prognosis-Related and OVS-Related Coexpression Modules

Gene significance (GS) was used to measure the correlation between genes and traits, and an average GS value of all genes included in each coexpression module was used to calculate module significance (ME). The most significant module correlated with prognosis and OVS was selected for further analyzed.

**Figure 2:**
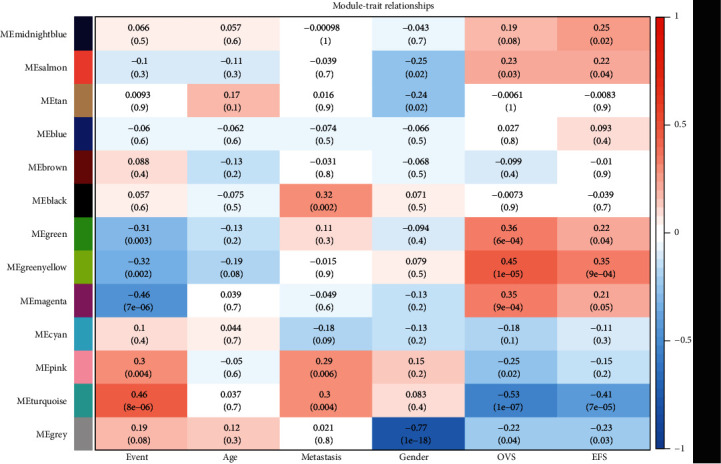
Heatmap of the correlation between modules genes and clinical traits. *x*-axis corresponds to the clinical features and *y*-axis to the identified modules. The color scale (blue to red) indicates correlation, top row: the correlation coefficient and bottom row: the *P* value. Event: death or alive; OVS: overall vital survival; EFS: event-free survival.

According to the module-trait relationship heatmap (Figure 2), the turquoise module was significantly correlated with the event (*r*^2^ = 0.46; *P* = 8*e* − 06), OVS (*r*^2^ = −0.53; *P* = 1*e* − 07), and EFS (*r*^2^ = −0.41; *P* = 7*e* − 05). Consequently, we focused on turquoise module in our further study.

### 3.3. PPI Network Construction and Hub Gene Identification

**Figure 3:**
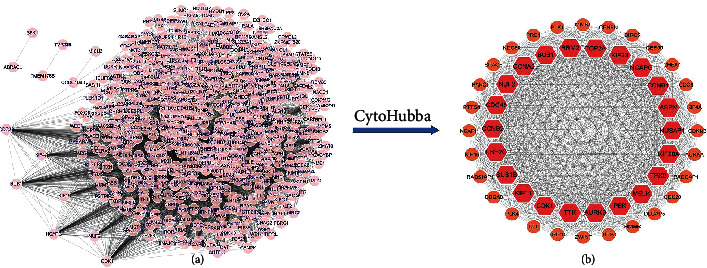
PPI network of turquoise module and identification of 50 hub genes. (a) Interaction network of all genes of turquoise module; (b) interaction network of 50 hub genes; the darker the color, the higher the MCC algorithm score.

Based on the STRING database, the PPI network of 880 genes in the turquoise module was constructed, and the top 50 hub genes in the network were selected using the MCC algorithm of cytoHubba (Figure 3).

### 3.4. Functional Enrichment of Hub Genes

**Figure 4:**
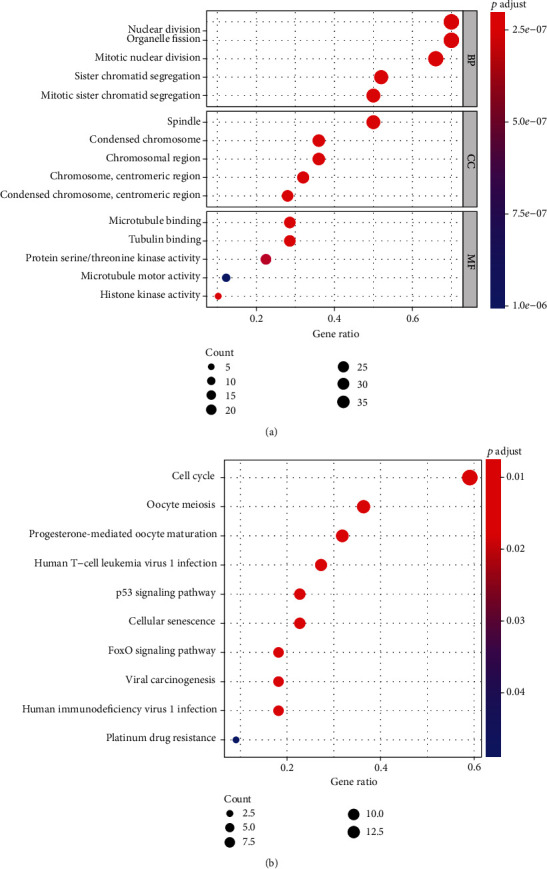
Functional enrichment of 50 hub genes. (a) GO enrichment in the 50 hub genes. (b) KEGG pathway enrichment in the 50 hub genes.

The Kyoto Encyclopedia of Genes and Genomes (KEGG) pathway and Gene Ontology (GO) enrichment analysis were used to further study the function of 50 hub genes. According to the GO analysis (Figure 4(a)), the 50 hub genes in the biological process (BP) were mainly enriched in nuclear division, organelle fission, mitotic nuclear division, sister chromatid segregation, and mitotic sister chromatid separation. The cellular component (CC) of 50 hub genes was mainly enriched in spindle, condensed chromosome, chromosomal region, chromosome (centromeric region), and condensed chromosome (centromeric region). And the molecular function (MF) of 50 hub genes was mainly enriched in microtubule binding, tubulin binding, protein serine/threonine kinase activity, microtubule motor activity, and histone kinase activity. Moreover, the 50 hub genes in the KEGG pathway enrichment analysis were mainly enriched in cell cycle, oocyte meiosis, progesterone-mediated oocyte maturation, human T-cell leukemia virus 1 infection, p53 signaling pathway, cellular senescence, FoxO signaling pathway, viral carcinogenesis, human immunodeficiency virus 1 infection, and platinum drug resistance (Figure 4(b)).

### 3.5. Establishment and Validation of a Risk Assessment Model

**Figure 5:**
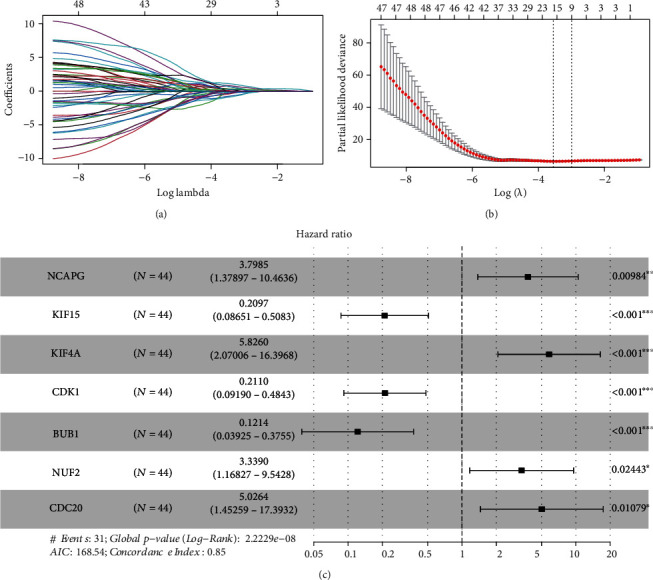
Identification of pivotal prognosis-related genes and establishment of a risk assessment model. (a) The trajectory of each independent variable. The horizontal axis shows the logarithm of lambda, and the vertical axis displays the coefficient of lambda. (b) The confidence interval under each lambda. (c) The forest map of the multivariate Cox analysis on the 7 independent prognostic feature genes.

**Figure 6:**
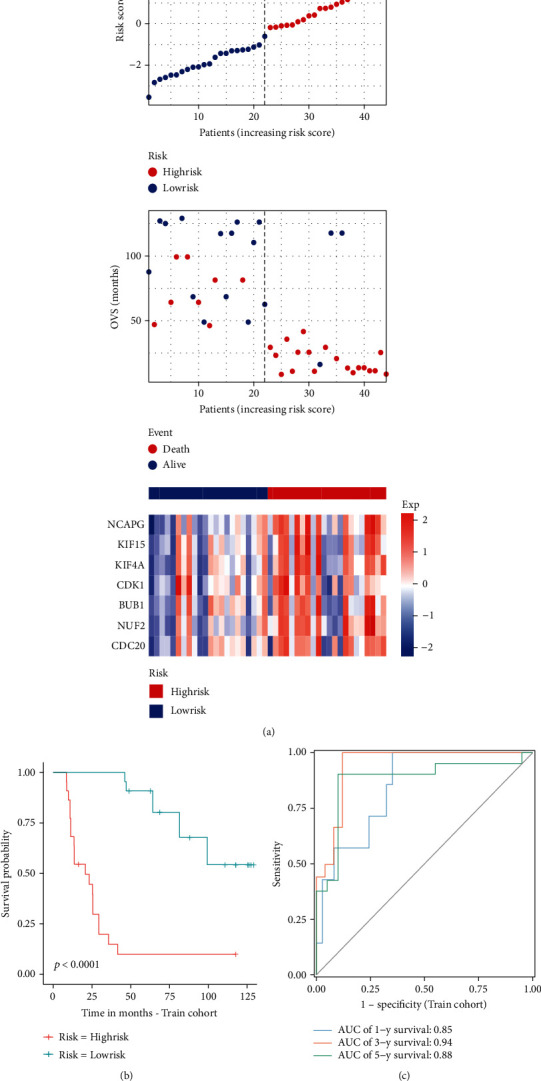
Prognostic analysis of the model in train cohort. (a) Distribution of the risk score, overall vital survival (OVS), and expression level of 7 pivotal genes in the model. (b) Kaplan-Meier survival analysis. (c) Time-dependent ROC curves of the prognostic model for the ES train cohort.

**Figure 7:**
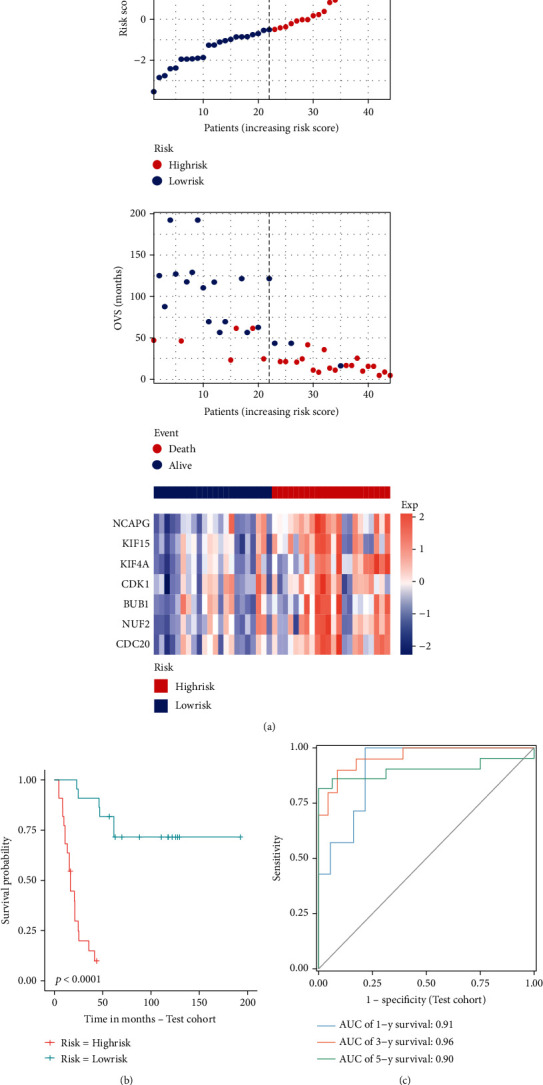
Validation of the efficacy of the risk model in the ES test cohort. The (a) risk score, survival status, gene expression heatmap, (b) Kaplan-Meier survival, and (c) time-dependent ROC curves of the prognostic model for the ES test cohort.

**Figure 8:**
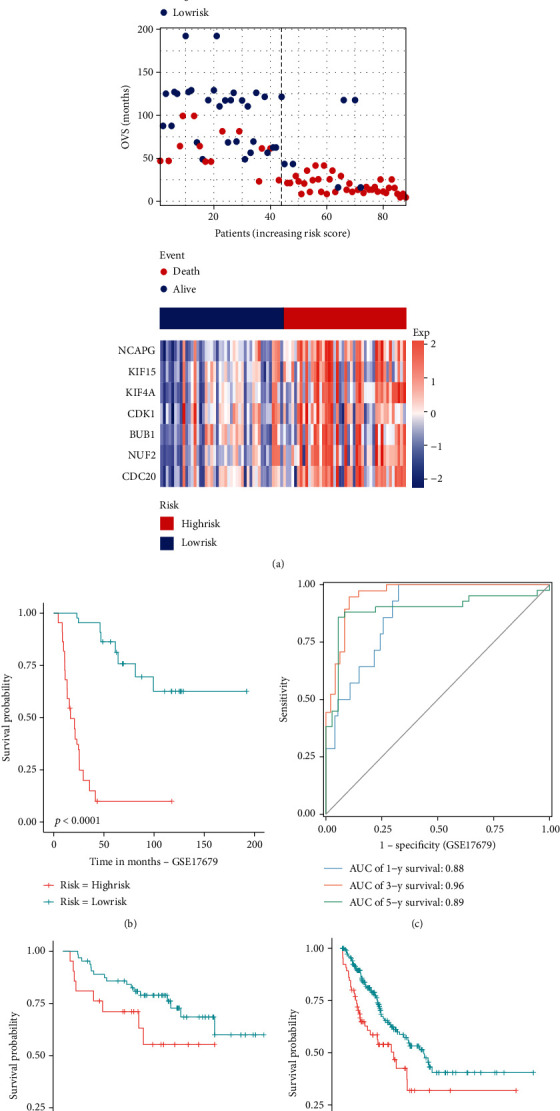
Validation of the efficacy of the risk model in the ES entire GSE17679 cohort, GSE63157, and TCGA-SARC. The (a) risk score, survival status, gene expression heatmap, (b) Kaplan-Meier survival, and (c) time-dependent ROC curves of the prognostic model for the entire GSE17679 cohort. And the Kaplan-Meier survival for (d) GSE63157-cohort and (e) TCGA-SARC.

We used univariate Cox regression analysis, LASSO regression analysis, and multivariate Cox regression analysis to screen out pivotal genes (Figures 5(a) and 5(b)). Then, we established an optimal risk assessment model based on seven pivotal genes including NCAPG, KIF15, KIF4A, CDK1, BUB1, NUF2, and CDC20 (Figure 5(c)). For verifying the accuracy of this model, the train cohort was divided into high-risk and low-risk subgroups based on the median risk score. As compared to the high-risk group, the survival of the low-risk subgroup was significantly longer (Figure 6(a)). As shown in Figure 6(b), the Kaplan-Meier analysis of two subgroups indicated that this model exhibited a statistically significant prognostic difference in ES (*P* value < 0.0001). And the AUC value of 1-, 3-, and 5-year survival was 0.85, 0.94, and 0.88, which indicated a good predictive accuracy of this model (Figure 6(c)). Next, we do the same process for test cohort and entire GSE17679. As shown in Figures 7(a)–7(c), the results of the test cohort are as follows: significantly different survival status, *P* value of Kaplan-Meier analysis <0.0001, and AUCs of ES at 1-, 3-, and 5-year were 0.91, 0.96, and 0.90. And as shown in Figures 8(a)–8(c), the results of entire CSE17679 set are as follows: significantly different survival status, *P* value of Kaplan-Meier analysis <0.0001, and AUCs of ES at 1-, 3-, and 5-year were 0.88, 0.96, and 0.89. Both of results indicated a good predictive accuracy of this model. Finally, the Kaplan-Meier analysis of GSE63157 is *P* value = 0.11 and for TCGA-SARC is *P* value = 0.004; although *P* value of GSE63157 was not significant, a similar prognostic trend was observed, so it revealed a good versatility and practicality of this model (Figures 8(d) and 8(e)).

### 3.6. Analysis of Each Single Pivotal Prognostic Gene

**Figure 9:**
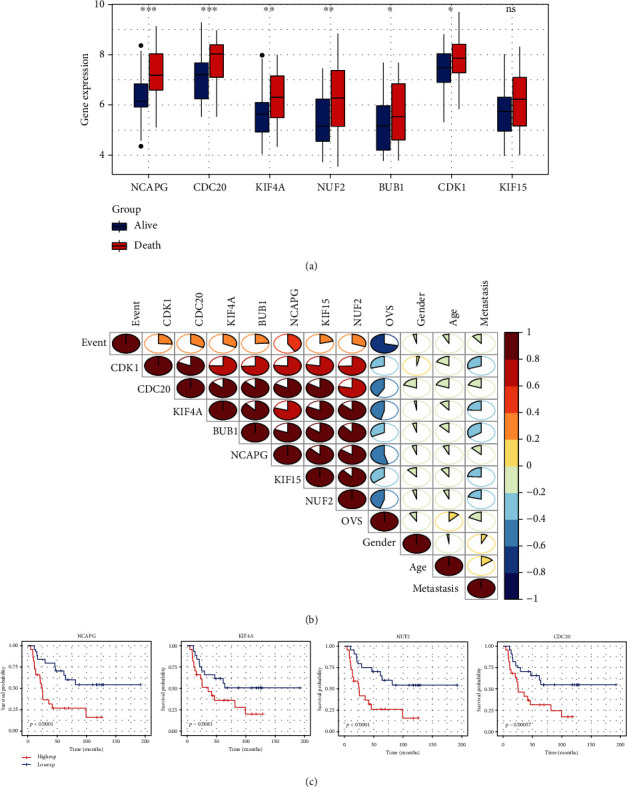
Analysis of each single pivotal prognostic gene. (a) The expression of each pivotal prognostic gene in death and alive subgroups. (b) The correlation analysis between the expression levels of each gene and clinical traits. (c) Kaplan-Meier survival analysis of four pivotal prognostic genes based on the median expression of each one.

According to the boxplot (Figure 9(a)), expression levels of 6 hub genes are clearly higher in the death subgroup than in the alive subgroup, except for KIF15. Then, we utilized the heatmap to reveal the correlations of seven pivotal genes with clinical traits. The result indicated all genes were significantly associated with OVS and event, and four genes (NCAPG, KIF4A, NUF2, and CDC20) may play a more important role in ES prognosis (Figure 9(b)). And, as shown in (Figure 9(c)), the results of the survival analysis also verified that these four key genes indeed play a more important role in prognosis. In conclusion, all of the results determined that the seven pivotal genes were statistically significant prognostic factors for ES, and four pivotal genes deserve further study.

### 3.7. Construction of the Nomogram and Validation

**Figure 10:**
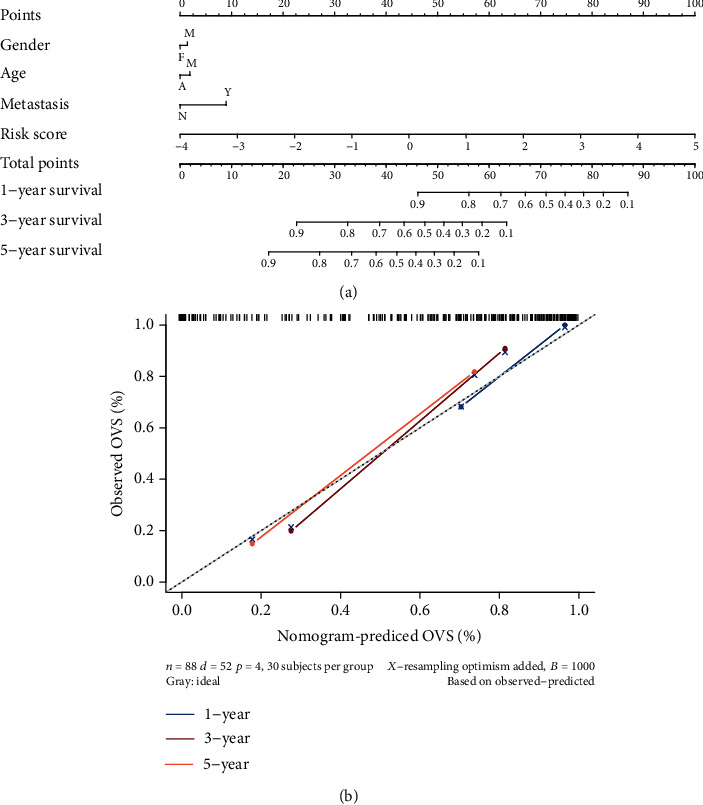
Nomogram for predicting the 1-, 3-, and 5-year survival probability of patients with ES. (a) Prognostic nomogram for ES patients. (b) Calibration curves for the nomogram at 1-, 3-, and 5-year.

At the end of this research, a nomogram was constructed incorporating risk score and clinical factors, which can help clinicians to predict the prognosis of patients based on their total points (Figure 10(a)). And the calibration curve of 1-, 3-, and 5-year survival (Figure 10(b)) indicated that this nomogram was accurate.

## 4. Discussion

As the second most common pediatric bone tumor, ES is characterized by pathognomonic FET-ETS gene fusions and is an invasive tumor with early metastatic spread, high recurrence, and low 5-year survival [5, 6, 23]. For ES, targeted therapy is still challenging to achieve, and no reliable statistical tool is available to estimate survivors' lifespans [4]. Hence, it is necessary to identify new target genes and develop a prognosis-related model, which can improve the prognosis of patients with ES.

In this study, we identified one module that was associated with OVS and event by using weighted coexpression analysis; then, 50 hub genes were selected by cytoHubba. The functional enrichment analysis revealed that 50 hub genes were enriched in the protein transcription, cell proliferation, and cell-cycle control. The result is consistent to FET/ETS family of biology function [24], so it is worth doing further study. Based on the three analysis methods (univariate Cox regression analysis, LASSO regression analysis, and multivariate Cox regression analysis), pivotal genes were identified. Combining pivotal genes with clinical traits, we finally established an optimal 7-gene-based risk assessment model. To verify the reliability of this model, the train cohort was divided into the high-risk group and low-risk group according to the median risk score, and the result indicates that the OVS of patients in the high-risk group was significantly shorter than that in the low-risk group. And the ROC curve (AUCs of 1-, 3-, and 5-year survival were 0.85, 0.94, and 0.88) and km-plot (*P* value < 0.0001) also indicated a good predictive accuracy for this model. Furthermore, the results of the other two cohorts (test and entire GSE17679) and two independent datasets were satisfactory, which also showed this model on a good predictive accuracy, versatility, and practicality.

In the 7 pivotal genes, every gene was clearly correlated with OVS and event, but four genes (NCAPG, KIF4A, NUF2, and CDC20) may play a more important role in ES prognosis. Therefore, further study of these four genes is warranted. Non-SMC condensing complex subunit G (NCAPG) is a crucial component of the condensing complex, which binds to chromosomes at start of mitotic division and dissociates from them when mitotic division ends. Previous studies have reported that NCAPG is a promising therapeutic target across different tumor types, and it may be associated with development and progression of hepatocellular carcinoma (HCC) [25, 26], esophageal squamous cell carcinoma (ESCC) [27], colorectal cancer (CRC) [28], breast cancer (BC) [29], prostate cancer (PCa) [30], kidney renal papillary cell carcinoma (KIRP) [31], endometrial cancer (EC) [32], bladder cancer (BLCA) [33], gastric cancer (GC) [34], glioblastoma (GBM) [35], and so on. And in the present study, we also find NCAPG was highly associated with the prognosis of ES; therefore, NCAPG may be a potential therapeutic target.

Kinesin family member 4A (KIF4A) was a new component of the chromosome segregation machinery and acted critical roles in mediating spindle organization and cytokinesis [36]. Previous studies have demonstrated the crucial role of KIF4A in the prognosis of several carcinomas, including colorectal cancer, cervical cancer, oral cancer, and lung cancer [37–39]. Although KIF4A has not been extensively studied in ES, the results of the current study showed that it may also be an important prognostic biomarker.

NUF2 component of NDC80 kinetochore complex (NUF2) is a component of a conserved protein complex associated with the centromere, and one study had demonstrated that NUF2 upregulation is a common feature of many cancers (TCGA-SARC dataset was also incorporated in this study) [40]. So, the prognostic potential and functional impact of NUF2 in ES warrants further studies.

Cell division cycle 20 (CDC20) regulates cell division and plays an important role in tumorigenesis and tumor progression. The upregulation of CDC20 is associated with poor prognosis of prostate cancer [41], breast cancer [42], and colorectal cancer [43]. In addition, CDC20 can contribute to cardiac hypertrophy by promoting LC3 degradation and inhibiting autophagy [44]. According to the results of study, CDC20 may also be a potential prognostic biomarker of ES.

Finally, a visual nomogram was created for quantitatively assessing the overall survival of ES patients using the risk score and clinical prognostic variables. And the results of the calibration curve suggest that the nomogram was dependable. Because of the rapid advances in genome sequencing, the cost of sequencing now is affordable for most patients. So, this nomogram can become a reliable and practical statistical tool, which can help clinician to evaluate the survival and prognosis of patients with ES.

## 5. Conclusions

This study established a 7-gene-based risk assessment model and demonstrated its good performance on predicting the prognosis of ES. And this study also found four pivotal genes may be the potential prognostic biomarker of ES. Although, based on three datasets, we detect significantly association between the predicted prognosis and the simulated risk score for ES patients, and this association has not yet been clinically confirmed. Therefore, the verification of this 7-gene risk assessment model and the research on the regulatory mechanism of single genes in model need to be further carried out. Taken together, this study provides a new method for evaluating the survival and prognosis of patients with ES and provides several potential therapeutic targets for ES.

## Data Availability

The data are available at Gene Expression Omnibus: https://www.ncbi.nlm.nih.gov/geo/ and The Cancer Genome Atlas Program (TCGA): https://www.cancer.gov/about-nci/organization/ccg/research/structural-genomics/tcga.

## Supplementary Materials

Supplementary 1 Supplemental Figure 1: check scale-free topology. The adjacency matrix was defined using soft-thresholds with *β* = 6.

Supplementary 2 Supplemental Figure 2: heatmap depicting the topological overlap matrix (TOM) among selected 400 genes based on coexpression modules. The light color represents a low overlap, and the progressively darker red color represents an increasing overlap.

Supplementary 3 Supplemental Figure 3: clustering dendrogram of samples with trait heatmap.
