# Supplementary figures and images for: Construction of a Prognosis-Related Gene Signature by Weighted Gene Coexpression Network Analysis in Ewing Sarcoma
Source: Comput Math Methods Med. 2022 Jan 27;2022:8798624. doi: 10.1155/2022/8798624 (PMC8814720; doi:10.1155/2022/8798624)

Histogram of k

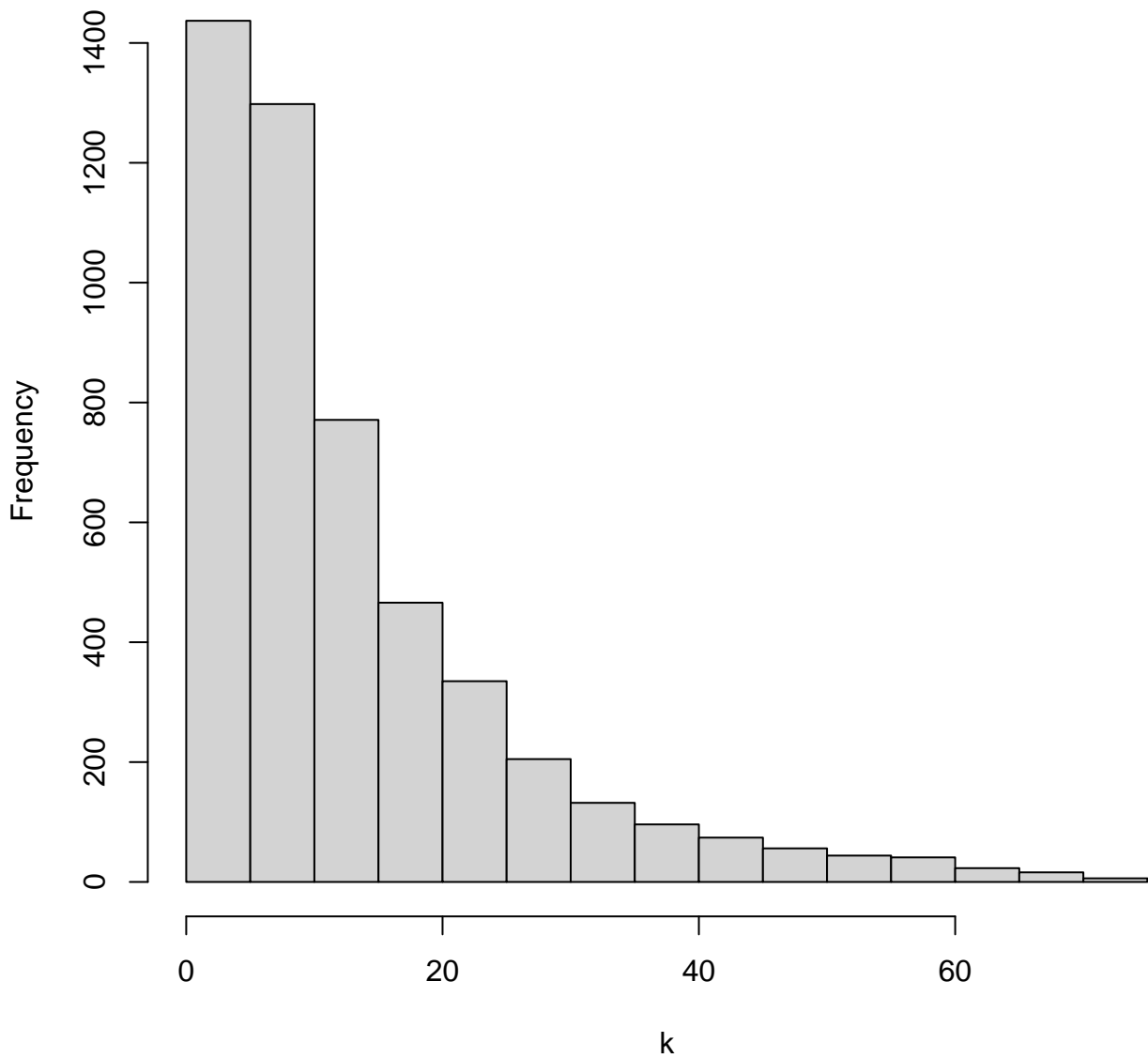

Check scale free topology scale  $R^2= 0.89$  , slope=  $-1.84$

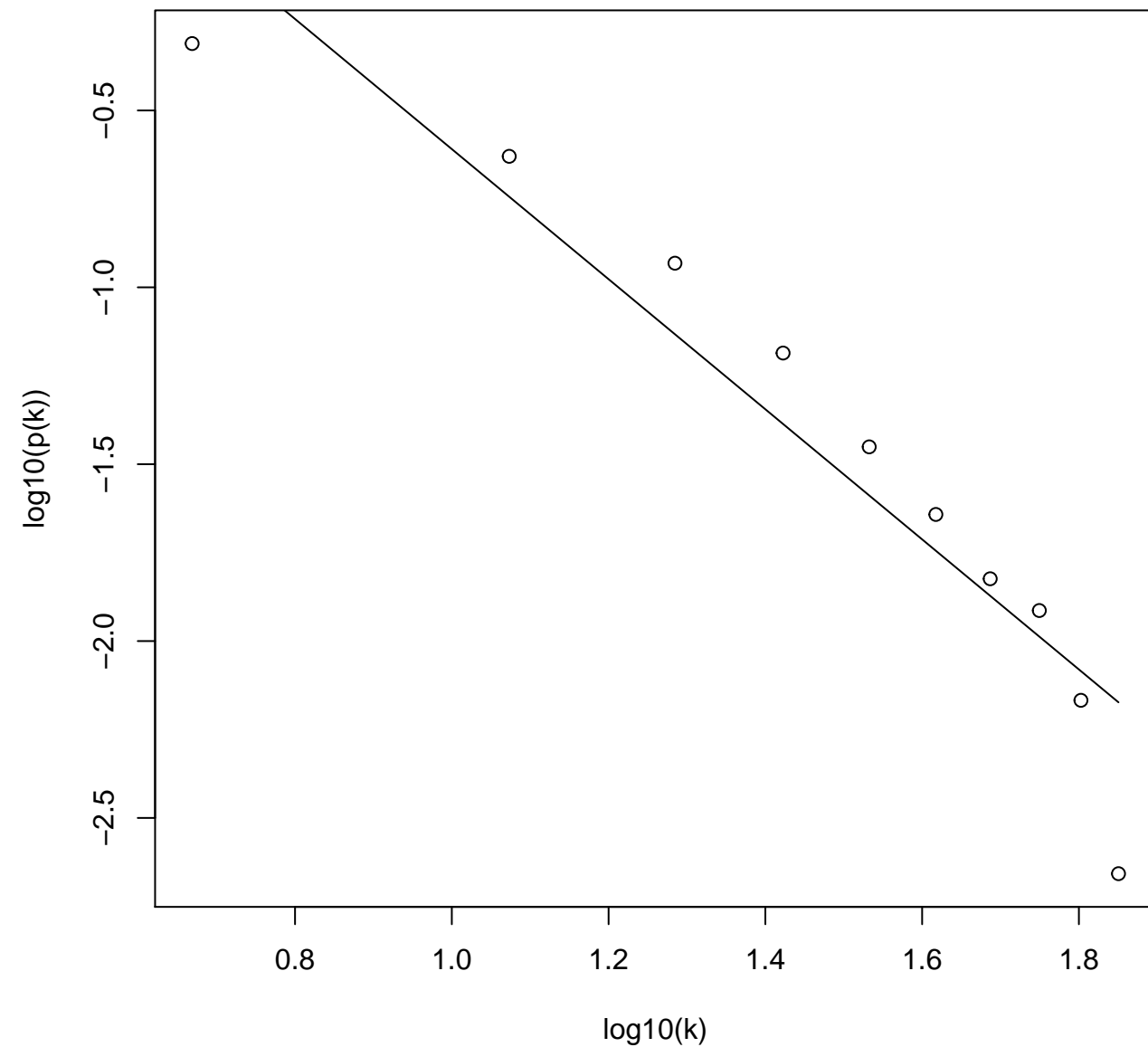

Supplement: Supplementary 1 — Supplemental Figure 1: check scale-free topology. The adjacency matrix was defined using soft-thresholds with β = 6. [file 8798624.f1.pdf]

Network heatmap plot, select 400 genes

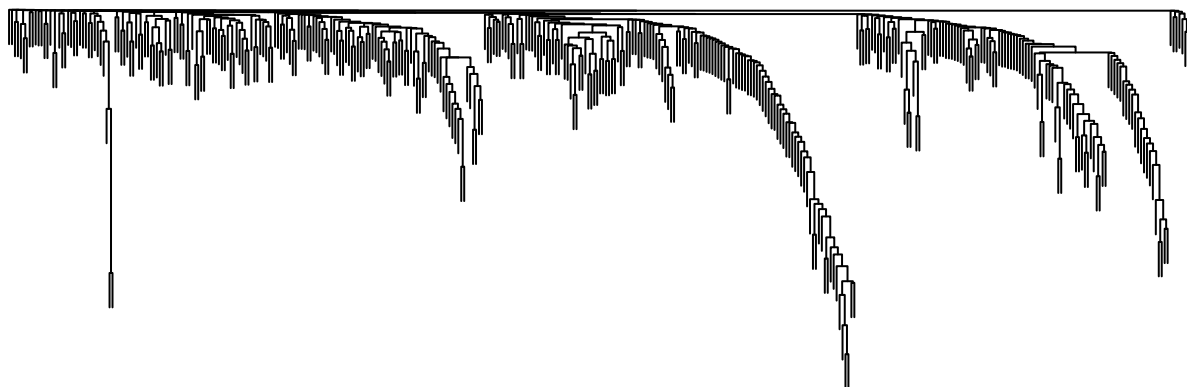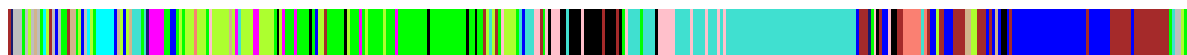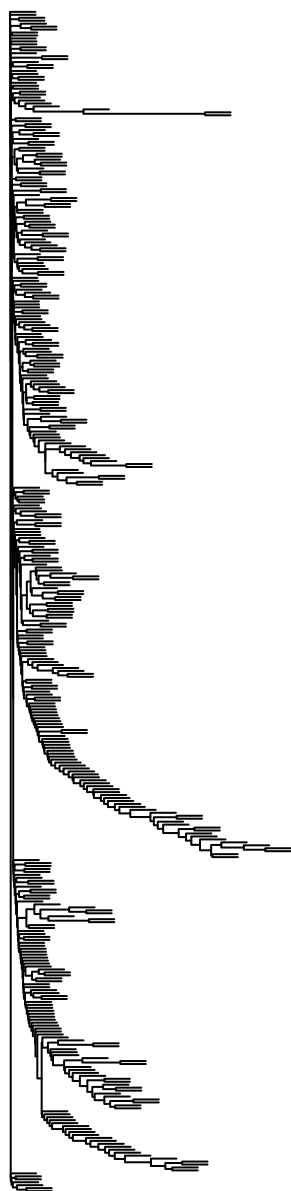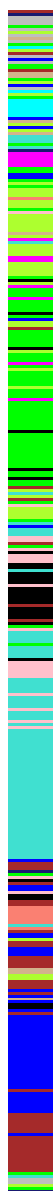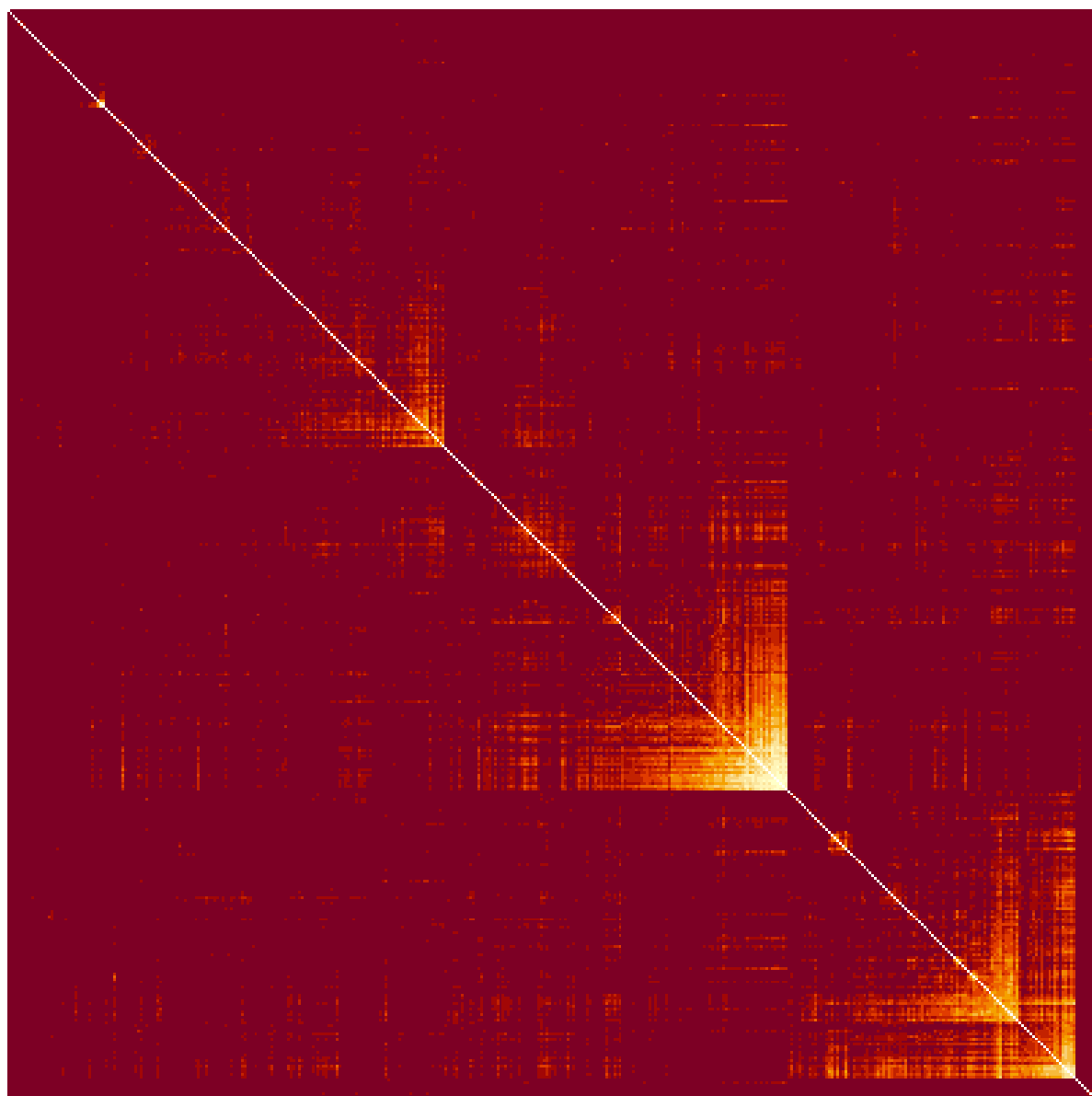

Supplement: Supplementary 2 — Supplemental Figure 2: heatmap depicting the topological overlap matrix (TOM) among selected 400 genes based on coexpression modules. The light color represents a low overlap, and the progressively darker red color represents an increasing overlap. [file 8798624.f2.pdf]

Sample dendrogram and trait heatmap

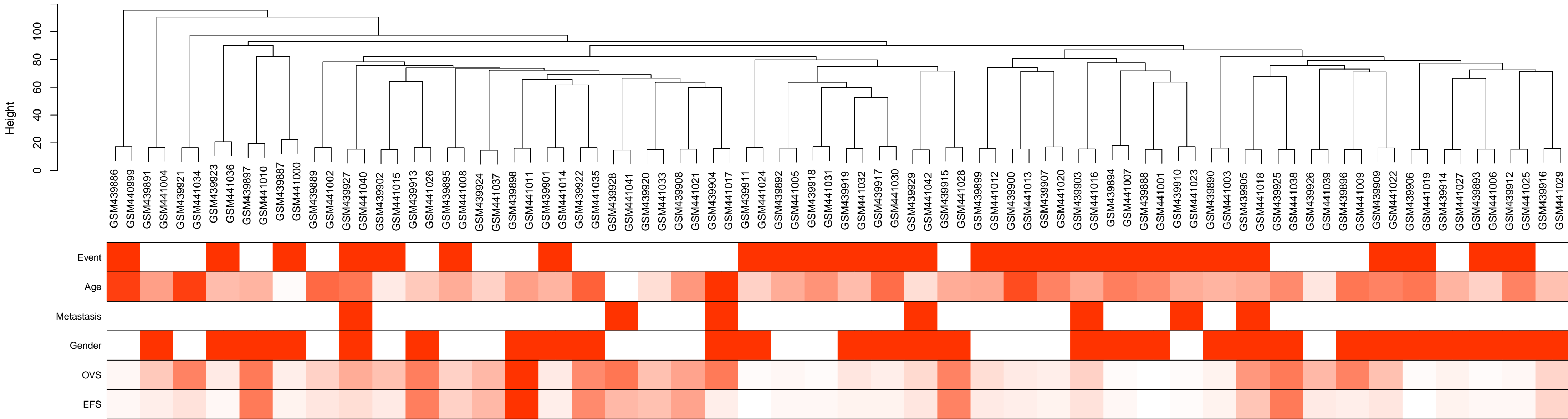

Supplement: Supplementary 3 — Supplemental Figure 3: clustering dendrogram of samples with trait heatmap. [file 8798624.f3.pdf]
